# METTL14 affects UVB‐induced human dermal fibroblasts photoaging via miR‐100‐3p biogenesis in an m^6^A‐dependent manner

**DOI:** 10.1111/acel.14123

**Published:** 2024-02-21

**Authors:** Lihao Chen, Yu Hu, Min Zhang, Lihao Liu, Jing Ma, Zhuohong Xu, Jiaan Zhang, Heng Gu, Kun Chen

**Affiliations:** ^1^ Jiangsu Key Laboratory of Molecular Biology for Skin Diseases and STIs, Institute of Dermatology, Chinese Academy of Medical Sciences and Peking Union Medical College Nanjing China

**Keywords:** ERRFI1, human dermal fibroblasts photoaging, m^6^A modification, METTL14, miR‐100‐3p

## Abstract

Exposure to ultraviolet radiation can lead to skin photoaging, which increases the risk of skin tumors. This study aims to investigate how microRNA m^6^A modification contributes to skin photoaging. This study found that skin fibroblasts exposed to a single UVB dose of 30 mJ/cm^2^ exhibited characteristics of photoaging. The m^6^A level of total RNA decreased in photoaged cells with a down‐regulated level of METTL14, and overexpression of METTL14 displayed a photoprotective function. Moreover, miR‐100‐3p was a downstream target of METTL14. And METTL14 could affect pri‐miR‐100 processing to mature miR‐100‐3p in an m^6^A‐dependent manner via DGCR8. Furthermore, miR‐100‐3p targeted at 3′ end untranslated region of ERRFI1 mRNA with an inhibitory effect on translation. Additionally, photoprotective effects of overexpression of METTL14 were reversed by miR‐100‐3p inhibitor or overexpression of ERRFI1. In UVB‐induced photoaging of human skin fibroblasts, METTL14‐dependent m^6^A can regulate miR‐100‐3p maturation via DGCR8 and affect skin fibroblasts photoaging through miR‐100‐3p/ERRFI1 axis.

## Graphical abstract

Upon photoaging in human dermal fibroblasts, the expression of methyltransferase METTL14 decreases, leading to reduced m^6^A modification levels on pri‐miR‐100. This reduction hinders the binding of DGCR8, obstructing the maturation of pri‐miR‐100, subsequently diminishing the expression of miR‐100‐3p, and ultimately causing the upregulation of downstream ERRFI1 expression.

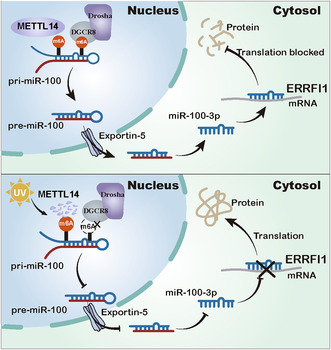

**Abbreviations**
3′‐UTR: 3′‐untranslated region
ALKBH5: AlkB homolog 5
CCK‐8: Cell Counting Kit‐8
CPDs: cyclobutane pyrimidine dimers
DEGs: Differentially expressed genes
DGCR8: DiGeorge syndrome critical region 8
DMEM: Dulbecco's modified eagle's medium
ECL: Enhanced chemiluminescence
EGFR: Epidermal growth factor receptor
EPs: Endogenous photosensitizers
ERRFI1: ERBB receptor feedback inhibitor 1
FBS: Fetal Bovine Serum
Fc: Fold chage
FTO: Fat mass and obesity‐associated protein
GEO: Gene expression omnibus
GSE: GEO series
HRP: Horseradish peroxidase
IgG: Immunoglobulin G
m^6^A: N6‐methyladenenosine
MeRIP: Methylated RNA immunoprecipitation
METT14: Methyltransferase‐like 14
METTL3: Methyltransferase like‐3
miRNA: microRNA
mRNA: messenger RNA
Mut: Mutated type
ncRNA: Non‐coding RNA
PBS: Phosphate buffered saline
PMSF: Phenylmethylsulfonyl fluoride
pri‐miRNA: Primary miRNA
PVDF: Polyvinylidene difluoride
Rb: Retinoblastoma
RIP: RNA Immunoprecipitation
RIPA: Radioimmunoprecipitation assay buffer
UVB: Ultraviolet B
UVR: Ultraviolet radiation
WT: Wild type
WTAP: Wilms tumor 1 associated protein

## 1 INTRODUCTION

Aging is an ongoing and significant challenge for human beings, with skin aging being one of the most extensively studied areas within this field. Skin aging is a gradual natural process that is influenced by numerous intrinsic (genetic) as well as extrinsic (environmental) factors. The most significant of these environmental factors is long‐term exposure to ultraviolet radiation (UVR) from sunlight, which contributes to the development of photoaging. Consequently, it is crucial to prevent and treat photoaging. However, the underlying mechanisms that drive photoaging are not yet fully comprehended, thus it is of utmost importance to acquire a more profound comprehension of the underlying molecular mechanisms that govern the progression of skin photoaging.

Currently, over 150 types of post‐transcriptional modifications have been identified on both mRNA and ncRNA. Following its discovery in 1970, N6‐methyladenosine (m^6^A) has been recognized as the most widespread and abundant mRNA modification in eukaryotic cells (Boccaletto et al., 2022; Sun et al., 2019). The reversible and dynamic regulation of m^6^A modification suggests its potential importance in various biological functions. m^6^A modification has been demonstrated to occur on nearly all RNA species, including microRNA (miRNA), long non‐coding RNA (lncRNA), circular RNA (circRNA), small nuclear RNA (nsRNA), ribosomal RNA (rRNA), and various others (Huang et al., 2020). The methyltransferases (“Writers”), such as METTL3, METT14, METTL16, and WTAP, regulate the abundance of m^6^A modifications on target RNA, while demethylases (“Erasers”), including FTO and ALKBH5, remove them, allowing for dynamic regulation. Importantly, “readers” are a group of binding proteins that recognize and interact with m^6^A‐modified sites on RNA and exert various biological functions depending on their specific mode of interaction. For example, YTHDF1‐mediated binding to m^6^A‐modified RNA enhances its transcriptional output, whereas YTHDF2 facilitates its degradation (Deng et al., 2022).

RNA m^6^A methylation modification has been shown to regulate the DNA damage response induced by UV radiation. Specifically, in the context of the UV‐induced DNA damage response, RNA at the site of DNA damage rapidly acquires m^6^A modifications within 2 min, which enhances DNA damage repair and is jointly regulated by METTL3 and FTO. Notably, deficiency of the methyltransferase METTL3 results in a significant delay in DNA damage repair in damaged cells (Xiang et al., 2017). In addition, it has been revealed that exposure to medium‐wavelength UV (ultraviolet B, UVB) induces degradation of METTL14 via autophagy in HaCat cells, a human keratinocyte cell line. This degradation leads to an overall decrease in m^6^A levels in these cells. However, overexpression of METTL14 restores m^6^A levels and enhances DNA damage repair (Yang et al., 2021). These findings shed light on the important regulatory role played by RNA m^6^A methylation modification in UV‐induced damage, providing a basis for further exploration of the mechanisms underlying the role of RNA m^6^A modification in skin photoaging.

Recent research has demonstrated the involvement of m^6^A in miRNA biogenesis. Tea Berulava and colleagues performed RNA sequencing analysis on HEK293 cells after FTO demethylase knockdown and found that 249 miRNAs were upregulated while 35 miRNAs were downregulated. They confirmed the presence of m^6^A modifications on specific miRNAs using RNA immunoprecipitation experiments with anti‐m^6^A antibodies (Berulava et al., 2015). Claudio R and colleagues later showed that the methyltransferase METTL3 modifies pri‐miRNA with m^6^A, enhancing its recognition by DGCR8 and promoting processing into mature miRNAs. Deficiency of METTL3 reduces levels of DGCR8 bound to pri‐miRNA and total mature miRNA content while accumulating unprocessed pri‐miRNA (Alarcón et al., 2015). Moreover, studies have shown that METTL3‐mediated m^6^A modification promotes the maturation of target miRNAs in tumor cells, affecting specific tumorigenic characteristics such as proliferation, invasion, metastasis, and drug resistance (Han et al., 2019; Pan et al., 2021). The present findings highlight the pivotal role enacted by METTL3‐mediated m^6^A modification in miRNA biogenesis, indicating its potential usefulness as a promising therapeutic target for cancer.

The focus of this study was the determination of the role played by m^6^A modification in photoaged human dermal fibroblasts (HDFs) and the elucidation of the underlying mechanism responsible for how m^6^A modification contributes to the progression of HDFs photoaging. We first established a HDFs photoaging model using UVB irradiation at 30 mJ/cm^2^ and observed that both m^6^A modification and METTL14 were downregulated in photoaged HDFs. Overexpression of METTL14, however, displayed photoprotective effects. Subsequently, we identified miR‐100‐3p as the target of METTTL14 and discovered that METTL14 overexpression increased miR‐100‐3p expression levels through its ability to modulate DGCR8 binding to primary miR‐100 (pri‐miR‐100) via an m^6^A‐dependent manner. Finally, we were able to confirm that METTL14 affects HDFs photoaging through the miR‐100‐3p/ERBB receptor feedback inhibitor 1 (ERRFI1) axis. These findings provide significant insights into potential therapeutic targets for skin photoaging and highlight the regulatory role of m^6^A modification in HDFs photoaging.

## 2 RESULTS

### 2.1 UVB 30 mJ/cm^2^ radiation was used to induce HDFs photoaging

After 24 h of exposure, HDFs exhibited inhibited cell proliferation and morphological changes, including flattening, poor refraction, enlarged nuclei, and increased cell volume. Senescence‐associated β‐galactosidase staining revealed more positively stained cells in the UVB group compared to the control group (Figure S1a,b). Cell viability in the UVB group was significantly lower than that of the control group, as indicated by the CCK‐8 assay (Figure S1c). Western blotting analysis revealed upregulation of p53 and p21, hallmarks of cell senescence, and downregulation of type I collagen expression (Figure S1d). In addition, cell cycle analysis showed cell cycle arrest, with an enrichment in the G0/G1 phase and a reduction in the G2/M and S phases (Figure S1e,f). These findings confirm the successful establishment of a photoaging cellular model using UVB irradiation (30 mJ/cm^2^) of HDFs.

### 2.2 RNA m^6^A modification and METTL14 were downregulated in photoaged HDFs

**FIGURE 1:**
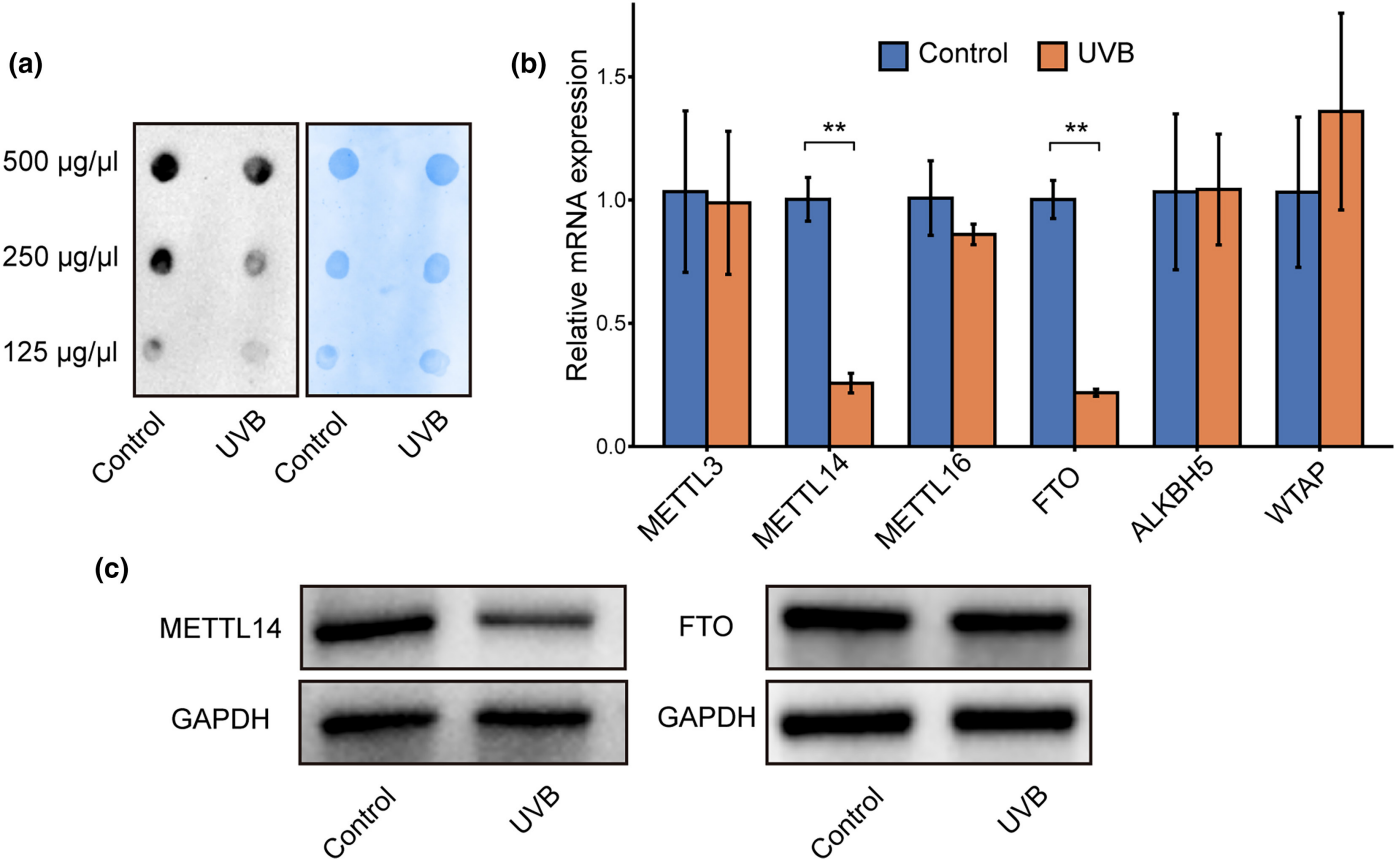
RNA m^6^A modification and METTL14 were downregulated in photoaged HDFs. (a) Dot blot analysis was used to determine total RNA m^6^A level in photoaged human dermal fibroblasts (HDFs), and methylene blue (MB) staining served as a loading control. (b) The mRNA level of common m^6^A‐regulated genes was evaluated by RT‐qPCR. (c) Western blot of protein level of METTL14 and FTO. All data are the mean ± SD. **p < 0.01. The significance of differences was determined with Student's *t* test.

To investigate potential alterations in RNA m^6^A modification during HDFs photoaging, RNA dot blot assay was used to assess total RNA m^6^A levels. A significant decrease in m^6^A modification was observed in photoaged HDFs (Figure 1a). Additionally, key enzymes regulating m^6^A modification, METTL14 and FTO, were significantly downregulated in photoaged HDFs at the mRNA expression level (Figure 1b), which was further confirmed by Western blotting analysis showing a decrease in METTL14 protein levels (Figure 1c). These findings suggest that reduced RNA m^6^A modification may contribute to photoaging in HDFs, potentially through dysregulation of METTL14.

### 2.3 Overexpression of METTL14 displayed photoprotective effects

**FIGURE 2:**
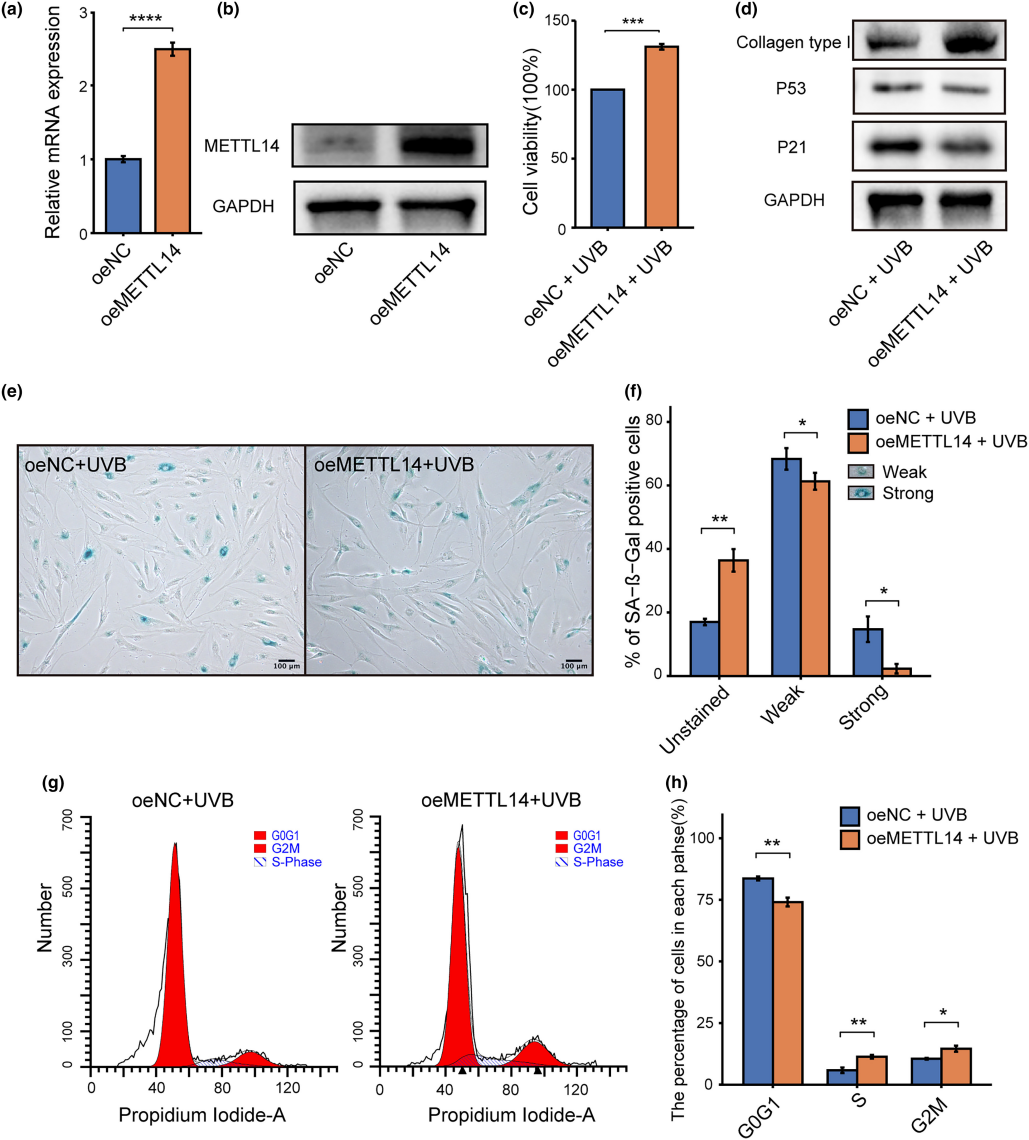
Overexpression of METTL14 could partially mitigate UVB‐induced HDFs photoaging. (a, b) RT‐qPCR and western blot of METTL14 in overexpression of METTL14 (oeMETTL14) by lentivirus in HDFs. (c) Cell counting kit‐8 (CCK‐8) was used to determine the cell viability of UVB‐induced photoaging of oeMETTL14 and oeNC HDFs. (d) Western blot analysis of collagen type I, p53 and p21 expression of oeMETTL14 and oeNC HDFs after UVB‐induced photoaging. (e) Senescence‐associated β‐galactosidase staining (SA‐β‐Gal) was used to identify photoaged cells of oeMETTL14 and oeNC HDFs after UVB‐induced photoaging. SA‐β‐Gal‐positive cells were dyed blue under optical microscopy. (f) SA‐β‐Gal‐positive cells were classified into two groups: strong and weak, and percentage of each group were shown in bar chart. (g, h) Cell cycle analysis of oeMETTL14 and oeNC HDFs after UVB‐induced photoaging by flow cytometry. All data are the mean ± SD. *p < 0.05, **p < 0.01, ***p < 0.001, ****p < 0.0001. The significance of differences was determined with Student's *t* test.

We utilized LV‐METTL14 to upregulate METTL14 expression in HDFs (Figure 2a,b) and subsequently induced photoaging in the cells. We assessed the effects of METTL14 overexpression on photoaged HDFs using SA‐β‐Gal staining, CCK‐8 assay, Western blotting of aging markers, and cell cycle analysis. Our findings demonstrated that overexpression of METTL14 partially restored the reduction in cell viability induced after UVB irradiation (Figure 2c). Further, the upregulation of p21 and p53, as well as the degradation of Type I collagen caused by UVB irradiation, were inhibited by the overexpression of METTL14 (Figure 2d). Overexpression of METTL14 also decreased the number of senescent cells following UVB irradiation, as observed using SA‐β‐Gal staining (Figure 2e,f). In addition, METTL14 overexpression mitigated the cell cycle disruption induced by UVB irradiation (Figure 2g,h). However, overexpression of METTL14 alone did not affect the aging phenotypes of HDFs as shown in Figures S2. Overall, our results suggest that overexpression of METTL14 can exert photoprotective effects against UVB‐induced HDFs photoaging.

### 2.4 METTL14‐dependent m^6^A modification modulated the maturation of pri‐miR‐100 via DGCR8

**FIGURE 3:**
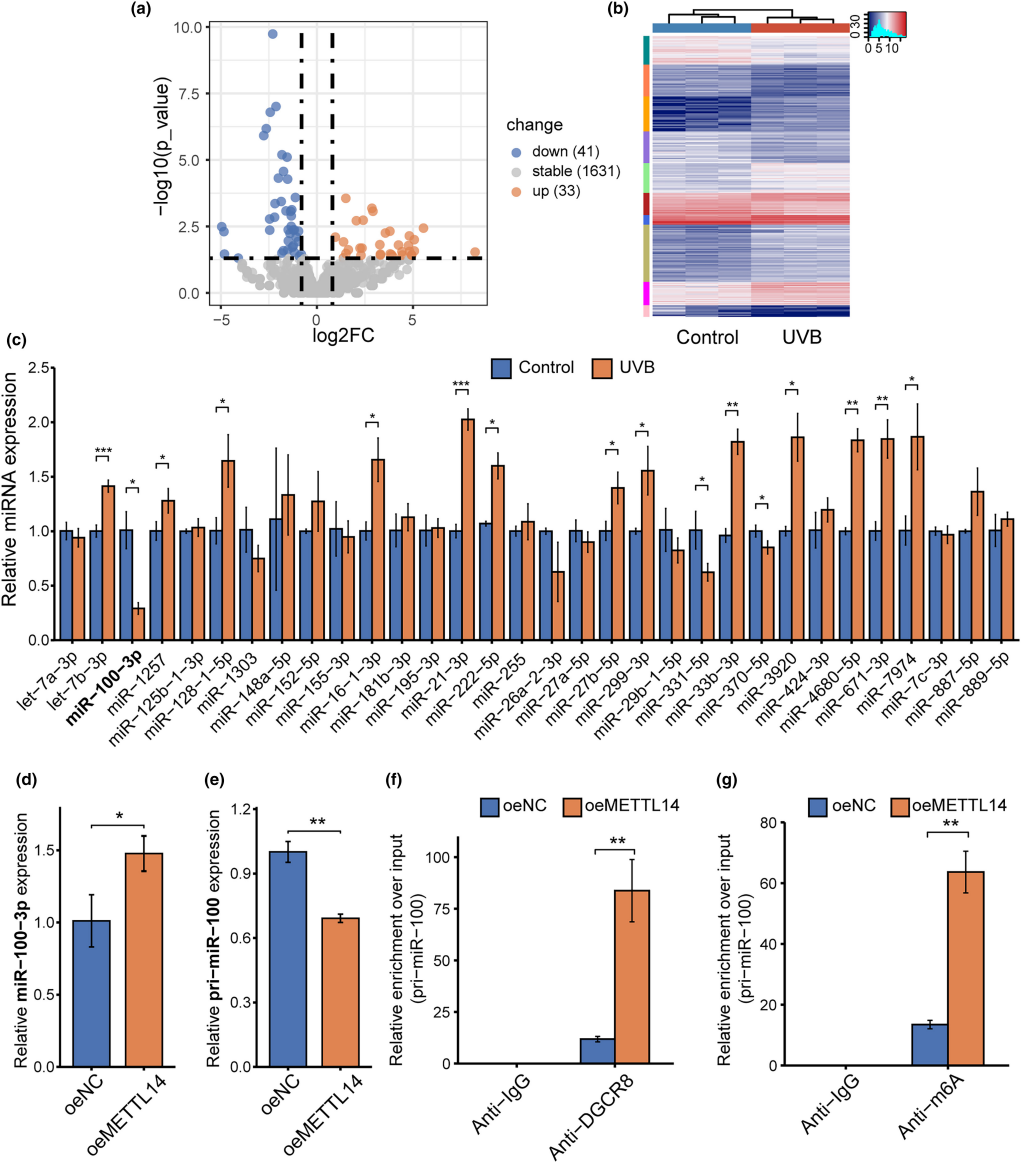
METTL14‐dependent m^6^A modification modulated the maturation of pri‐miR‐100 via DGCR8. (a) Volcano plot of miRNA sequencing data of UVB‐induced photoaged and control HDFs. Differentially expressed genes (DEGs) were shown by red (upregulated) and blue (downregulated) dots. DEGs screening criteria:*p* < 0.05, log2(Fc) ≥ 1.5. (b) Heatmap of DEGs. (c) RT‐qPCR validation of downregulated miRNAs in sequencing data. (d) RT‐qPCR of miR‐100‐3p in oeMETTL14 and oeNC HDFs. (e) RT‐qPCR of pri‐miR‐100 in oeMETTL14 and oeNC HDFs. (f) Immunoprecipitation of DGCR8‐binding RNA from oeMETTL14 and control HDFs followed by RT‐qPCR. (g) Immunoprecipitation of m^6^A‐modified RNA in oeMETTL14 and control HDFs followed by RT‐qPCR. All data are the mean ± SD. *p < 0.05, **p < 0.01, ***p < 0.001. The significance of differences was determined with Student's *t* test.

Recent studies have uncovered the crucial role of DGCR8 in pri‐miRNA processing and how it often necessitates METTL3 or METTL14 in different cancer types. In this study, miRNA sequencing data from photoaged HDFs and control HDFs were differentially analyzed using the screening criteria of *p*‐value < 0.05 and log2(Fc) ≥ 1.5 (Figure 3a,b). We identified differentially expressed miRNAs with downregulated expression levels in photoaged HDFs and then validated them through RT‐qPCR. The findings revealed a significant decline in the expression levels of miR‐100‐3p in photoaged HDFs (Figure 3c). Notably, overexpression of METTL14 resulted in a significant increase in miR‐100‐3p expression levels, while the level of pri‐miR‐100 decreased correspondingly, suggesting the involvement of METTL14 in regulating miR‐100‐3p maturation (Figure 3d,e). Additionally, knockdown of METTL14 in HDFs using siRNAs, as depicted in Figures S3a,b, resulted in decreased expression of miR‐100‐3p and increased expression of pri‐miR‐100, as shown in Figure S3c,d. To explore the potential mechanism responsible for the effects of METTL14 on miR‐100‐3p maturation, we conducted immunoprecipitation of DGCR8 from both control HDFs and those that had experienced METTL14 overexpression, followed by detection of RNA levels of pri‐miR‐100 using RT‐qPCR. Our findings displayed a significant elevation in the expression levels of pri‐miR‐100 bound to DGCR8 within METTL14‐overexpressing HDFs (Figure 3f). Furthermore, we carried out m^6^A‐immunoprecipitation assays on RNAs extracted from both METTL14‐overexpressing and control HDFs to determine the effect of METTL14 on pri‐miR‐100. Our findings indicated that METTL14 overexpression induced an increase in the amount of pri‐miR‐100 modified by m^6^A (Figure 3g). These findings collectively suggest that METTL14 can facilitate miR‐100‐3p maturation by modulating m^6^A modifications on pri‐miR‐100, which subsequently enhances its binding with DGCR8.

### 2.5 miR‐100‐3p interference rescued the photoprotection induced by overexpression of METTL14 in in photoaged HDFs

**FIGURE 4:**
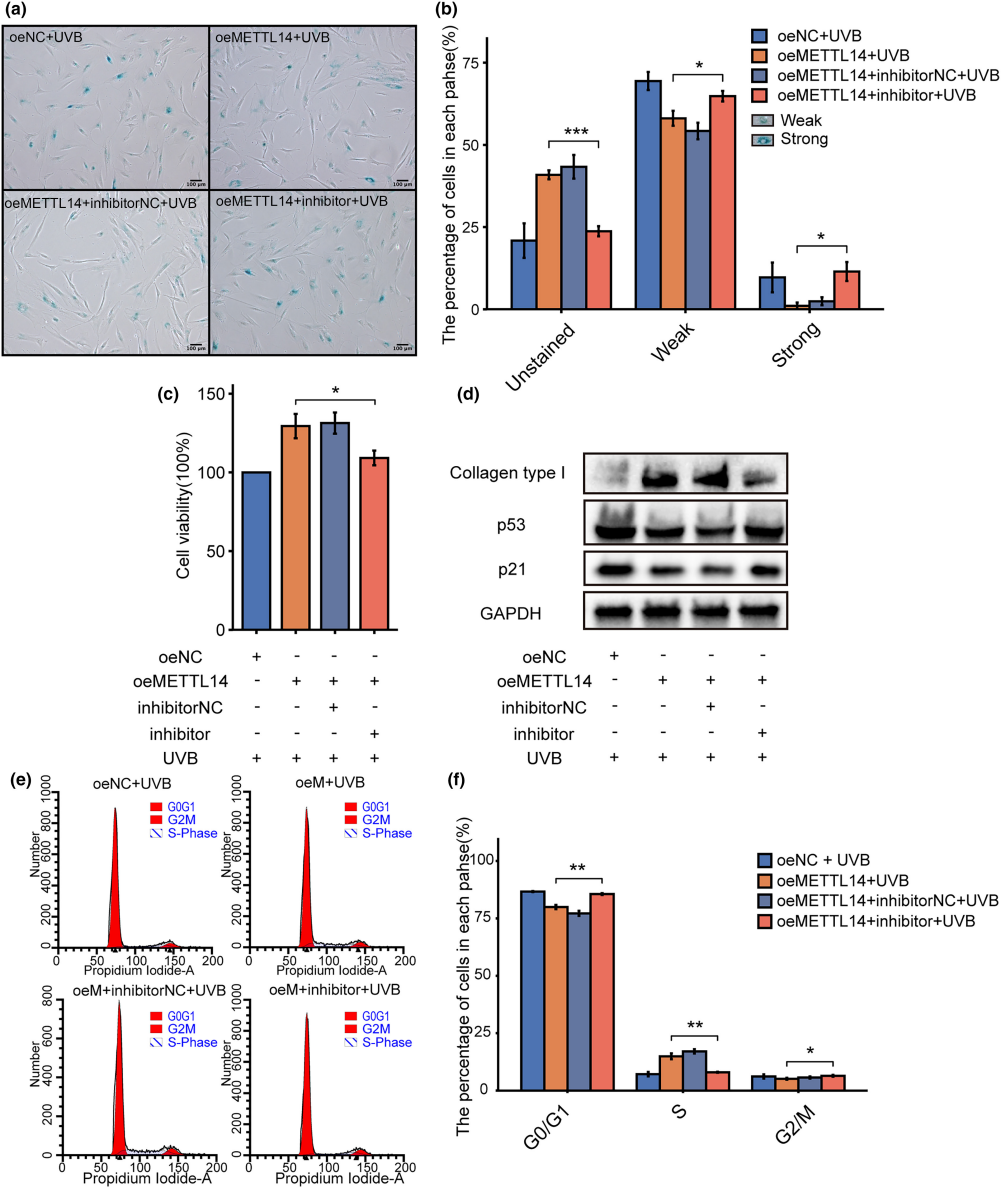
The miR‐100‐3p inhibition could block the photoprotective effects induced by overexpression of METTL14 in photoaged HDFs. (a) SA‐β‐Gal of UVB‐induced oeMETTL14 photoaged HDFs after treating with miR‐100‐3p inhibitor. SA‐β‐Gal‐positive cells were dyed blue under optical microscopy. (b) SA‐β‐Gal‐positive cells were classified into two groups: strong and weak, and percentage of each group were shown in bar chart. (c) Cell viability of UVB‐induced oeMETTL14 photoaged HDFs after treating with miR‐100‐3p inhibitor by CCK‐8. (d) Western blot analysis of collagen type I, p53 and p21 expression in UVB‐induced oeMETTL14 photoaged HDFs after treating with miR‐100‐3p inhibitor. (e, f) Cell cycle analysis of UVB‐induced oeMETTL14 photoaged HDFs after treating with miR‐100‐3p inhibitor by flow cytometry. *p < 0.05, **p < 0.01, ***p < 0.001. The significance of differences was determined with Student's *t* test.

After confirming the photoprotective effects of METTL14, we aimed to investigate its potential mediation via miR‐100‐3p. To this end, we treated cells overexpressing METTL14 with a miR‐100‐3p inhibitor and monitored the changes in the photoaging phenotype. SA‐β‐Gal staining revealed that the miR‐100‐3p inhibitor blocked the reduction in the proportion of senescent cells induced by METTL14 overexpression (Figure 4a,b). The results obtained via CCK‐8 assays indicated that the miR‐100‐3p inhibitor exerted an inhibitory impact on the restoration of cell viability, which had been previously induced via METTL14 overexpression (Figure 4c). Additionally, Western blotting showed that the miR‐100‐3p inhibitor prevented the decline of p21 and p53 levels, as well as the restoration of Type I collagen levels induced by METTL14 overexpression (Figure 4d). Furthermore, cell cycle analysis indicated that the miR‐100‐3p inhibitor impeded the alleviation of cell cycle arrest induced by METTL14 overexpression (Figures 4e,f). Overall, the results suggest that the photoprotective effects of METTL14 are partly mediated through miR‐100‐3p.

### 2.6 miR‐100‐3p targeted ERRFI1 in photoaged HDFs

**FIGURE 5:**
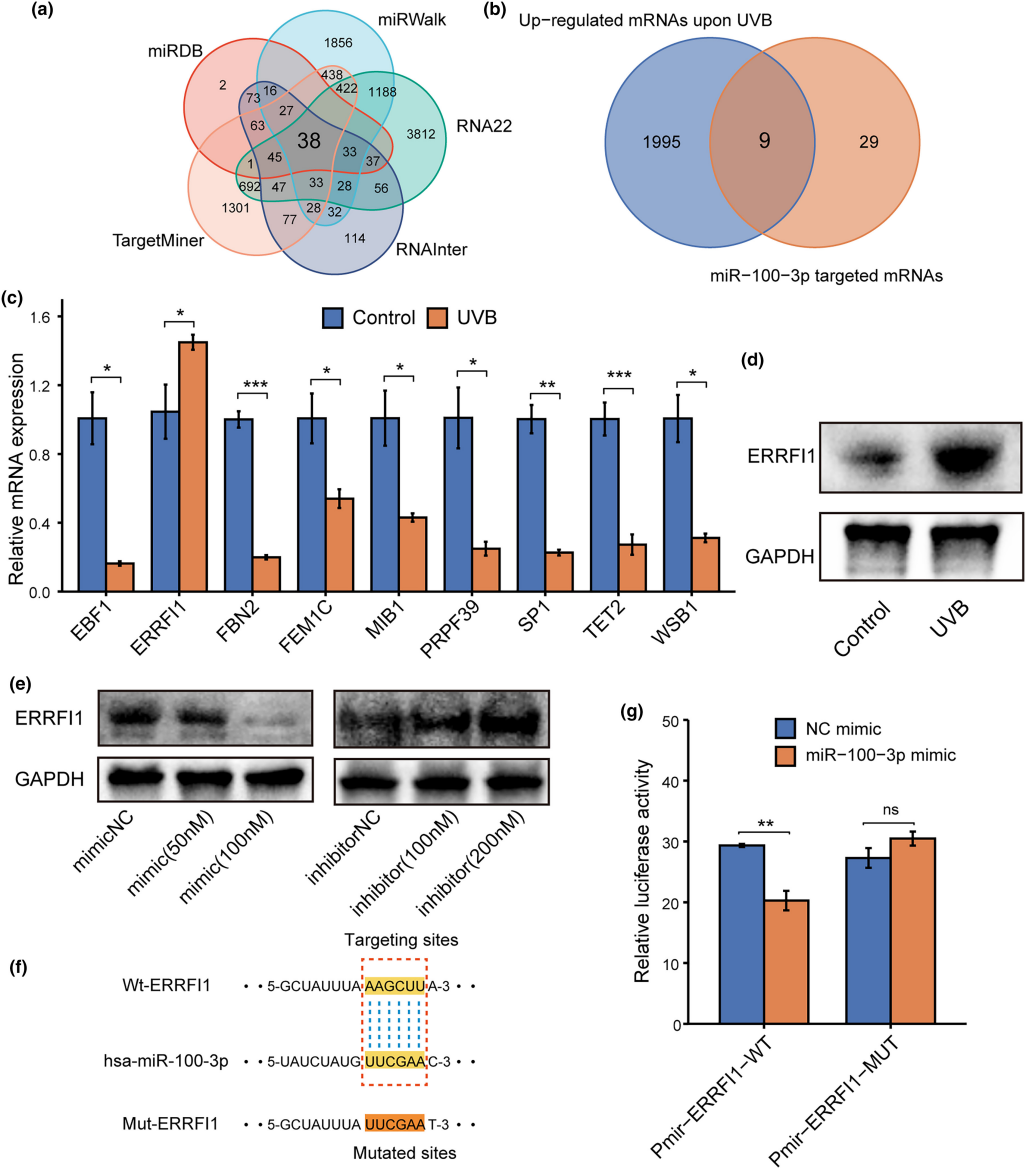
The miR‐100‐3p targeted ERRFI1 in photoaged HDFs. (a) miR‐100‐3p targeted mRNA prediction by miRDB, miRWalk, Targetminer, RNAinter and RNA22 databases. (b) Intersection of upregulated mRNAs of photoaged HDFs in GSE119009 dataset from GEO and predicted miR‐100‐3p targeted mRNAs. (c) RT‐qPCR validation of predicted miR‐100‐3p targeted mRNAs in photoaged HDFs. (d) Western blot analysis of ERRFI1 in photoaged HDFs. (e) Western blot analysis of ERRFI1 in HDFs after treated with miR‐100‐3p mimics and inhibitors. (f) The predicted binding wildtype (WT) sequence in 3′‐UTR of ERRFI1 for miR‐100‐3p and its corresponding mutant sequence (MUT) contained in luciferase vectors. (g) Luciferase reporter assay of 293 T cells co‐transfected with luciferase vectors containing WT or MUT ERRFI1 3′‐UTR sequence, along with miR‐100‐3p mimic or mimic NC. *p < 0.05, **p < 0.01, ***p < 0.001. The significance of differences was determined with Student's *t* test.

To investigate potential mRNA targets that could interact with miR‐100‐3p, we employed various prediction databases such as MiRDB, miRWalK, RNA22, TargetMiner, and RNAInter. Through this analysis, we identified a total of 38 candidate mRNAs (Figure 5a). To further narrow down our target genes, we utilized the GSE119009 dataset from GEO, which provides transcriptome sequencing results of UVB‐induced HDFs photoaging. We discovered 2004 upregulated mRNAs following UVB irradiation, of which only 9 overlapped with the 38 candidate mRNAs (Figure 5b). We then carried out RT‐qPCR on these candidate genes in photoaged HDFs and observed that only ERRFI1 was upregulated (Figure 5c). We then confirmed a significant increase in ERRFI1 protein expression through western blotting (Figure 5d). Through the use of miR‐100‐3p mimics and inhibitors in transfection, we established that miR‐100‐3p had a marked inhibitory effect on the protein expression of ERRFI1, while the miR‐100‐3p inhibitor could counter this effect (Figure 5e). Furthermore, luciferase reporter gene assays revealed that miR‐100‐3p can bind to the 3′‐UTR region of ERRFI1 mRNA and inhibit its translation leading to a decrease in fluorescence intensity. Notably, we observed that mutating the binding site sequence did not result in a change in fluorescence intensity (Figures 5f,g). These findings suggest that miR‐100‐3p plays an inhibitory role in ERRFI1 protein expression by targeting the 3′‐UTR region of ERRFI1 mRNA.

### 2.7 METTL14 regulated HDFs photoaging through miR‐100‐3p/ERRFI1 axis

**FIGURE 6:**
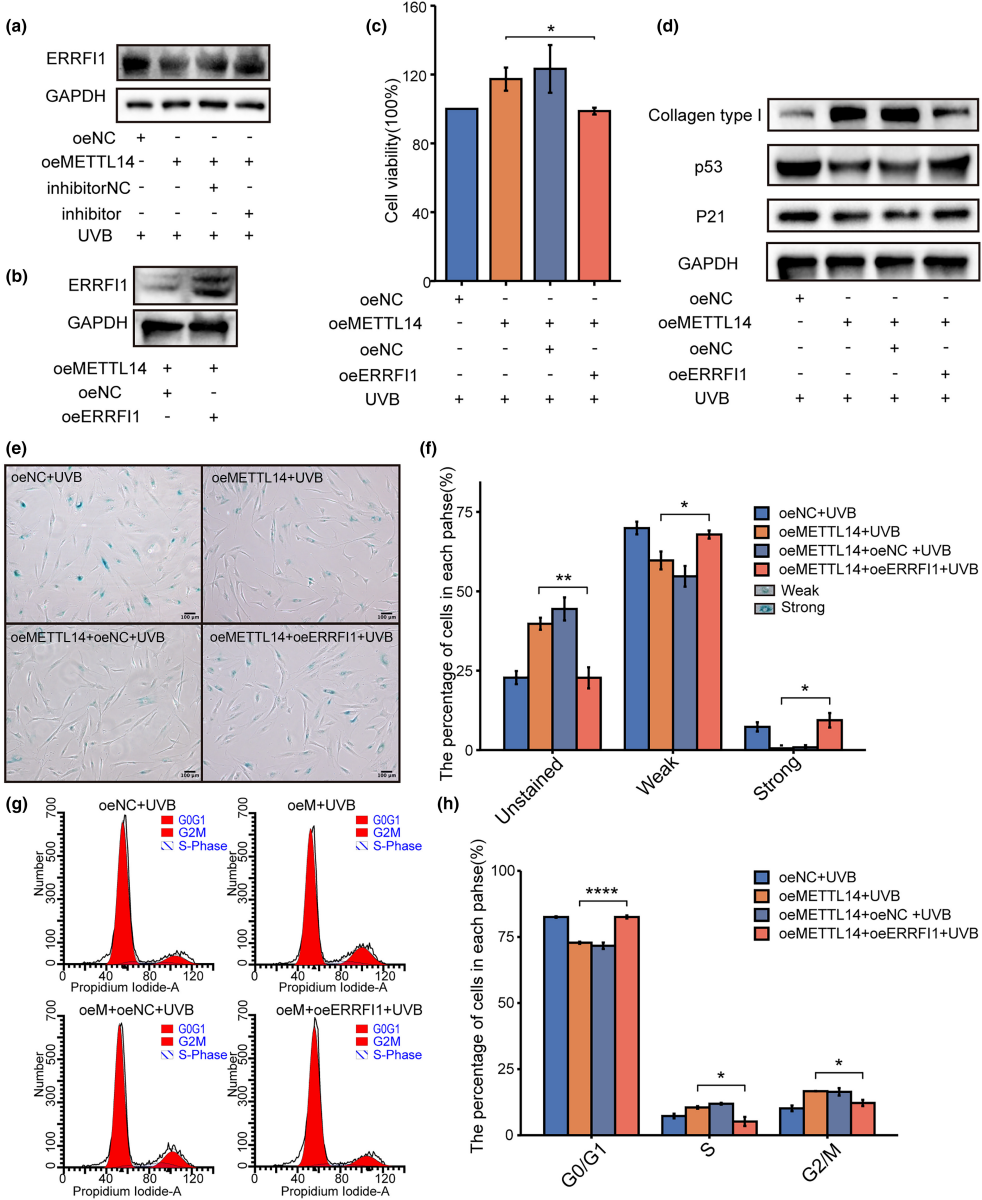
METTL14 regulated UVB‐induced HDFs photoaging through miR‐100‐3p/ERRFI1 axis. (a) Western blot analysis of ERRFI1 expression in UVB‐induced oeMETTL14 photoaged HDFs after treating with miR‐100‐3p inhibitor. (b) Western blot analysis of ERRFI1 expression in oeMETTL14 HDFs after transfection with overexpression plasmids containing ERRFI1 or alternatively, a blank vector. (c) Cell viability of UVB‐induced oeMETTL14 photoaged HDFs after transfection with overexpression plasmids containing ERRFI1 by CCK‐8. (d) Western blot analysis of collagen type I, p53 and p21 expression in UVB‐induced oeMETTL14 photoaged HDFs after transfection with overexpression plasmids containing ERRFI1. (e) SA‐β‐Gal of UVB‐induced oeMETTL14 photoaged HDFs after transfection with overexpression plasmids containing ERRFI1. SA‐β‐Gal‐positive cells were dyed blue under optical microscopy. (f) SA‐β‐Gal‐positive cells were classified into two groups: strong and weak, and percentage of each group were shown in bar chart. (g, h) Cell cycle analysis of UVB‐induced oeMETTL14 photoaged HDFs after t after transfection with overexpression plasmids containing ERRFI1 by flow cytometry. *p < 0.05, **p < 0.01, ****p < 0.0001. The significance of differences was determined with Student's *t* test.

Based on the results above, we propose a hypothesis that the methyltransferase METTL14 regulates photoaging via the miR‐100‐3p‐ERRFI1 axis. Overexpression of METTL14 resulted in a significant decrease in ERRFI1 protein expression levels, which was partially rescued by treatment with miR‐100‐3p inhibitors (Figure 6a). These findings suggest that METTL14 could regulate ERRFI1 expression via the miR‐100‐3p. To further elucidate the role of ERRFI1 in photoaging, we co‐overexpressed ERRFI1 along with METTL14 and evaluated its impact on the photoaging phenotype. We first confirmed the efficiency of ERRFI1 overexpression in oeMETTL14‐HDFs (Figure 6b). SA‐β‐Gal staining revealed that co‐overexpression of ERRFI1 could mitigate the reduction in the proportion of senescent cells caused by METTL14 overexpression (Figure 6c,d). CCK‐8 assays demonstrated that co‐overexpression of ERRFI1 also prevented the recovery of cell viability induced by METTL14 overexpression (Figure 6e). Furthermore, western blotting showed that co‐overexpression of ERRFI1 could impede the downregulation of p21 and p53, as well as the recovery of type I collagen levels induced by METTL14 overexpression (Figure 6f). Additionally, cell cycle analysis indicated that co‐overexpression of ERRFI1 could block the attenuation of cell cycle arrest caused by METTL14 overexpression (Figure 6g,h). Collectively, these findings suggest that METTL14 regulates photoaging via the miR‐100‐3p‐ERRFI1 axis.

### 2.8 Overexpression of ERRFI1 induced cellular senescence in HDFs

Given that ERRFI1 is a downstream target, our objective was to investigate its impact on HDFs. Consequently, we conducted ERRFI1 overexpression in HDFs (Figures S4a,b) to assess its impact on cellular senescence. The results of the CCK‐8 assay revealed that overexpression of ERRFI1 led to a decrease in cell viability. Western blot analysis demonstrated increased expression of p53 and p21, accompanied by a decrease in COL1 expression in the ERRFI1 overexpression group. The results of SA‐β‐Gal staining exhibited a higher proportion of senescent cells in the ERRFI1 overexpression group (Figures S4e,f). Cell cycle analysis revealed that overexpression of ERRFI1 resulted in cell cycle arrest at the G1 phase (Figures S4g,h). In summary, these results provide evidence that overexpression of ERRFI1 induces cellular senescence, suggesting its substantial involvement in cellular aging.

## 3 DISCUSSION

Photoaging of the skin manifests as various symptoms like coarse and fine wrinkles, dryness, increased fragility, abnormal pigmentation, and laxity (Bang et al., 2021), which can lead to photodamage‐induced carcinogenesis, including basal cell carcinoma, squamous cell carcinoma, and melanoma. The term ‘photoaging’ was first coined in the 1980s, and it is frequently used interchangeably with ‘dermatoheliosis’ (Kligman, 1989; Sayama et al., 2010). Ultraviolet (UV) radiation from photons absorbed by skin chromophores initiates photochemical reactions that contribute to photoaging (Martens et al., 2018; Trautinger, 2001). Therefore, establishing a reliable cellular model of photoaging is essential for scientific inquiry into this phenomenon. Previous studies have proposed multiple schemes for constructing photoaging cellular models. In this investigation, human dermal fibroblasts (HDFs) were induced using a 30 mJ/cm^2^ dose of UVB irradiation to construct a photoaging cellular model that mimics real‐life conditions and is experimentally convenient (Kang et al., 2020; Kim et al., 2019; Oh et al., 2018; Wang et al., 2022; Zhang et al., 2023).

This study demonstrates the feasibility of using UVB (30 mJ/cm^2^) to induce a skin fibroblast photoaging model from multiple perspectives. The SA‐beta Gal staining assay is a classic method used to confirm cell senescence, where β‐galactosidase is an acid hydrolase present in lysosomes that is usually active at pH 4.0, and under pH 6.0 conditions, it can be detected in senescent cells, known as SA‐beta Gal. SA‐beta Gal can catalyze X‐Gal to produce a deep blue product, which is detected in senescence‐specific beta‐galactosidase (Debacq‐Chainiaux et al., 2009). Cell senescence is defined as irreversible cell cycle arrest after losing the proliferative ability of the cell (Höhn et al., 2017). The proliferation of cells was assessed using CCK‐8 assay, and the cell cycle was detected using flow cytometry. The p53/p21 pathway plays a crucial role in regulating the senescence proliferation arrest processes and is often used as a senescence marker (Borrás et al., 2011; Rayess et al., 2012; Salama et al., 2014). Type I collagen is also a common skin aging marker. Therefore, we used Western blot experiment to detect the protein expression levels of type I collagen, p53, and p21. The results indicate that the number of SA‐beta Gal staining‐positive cells, cell viability, and cell cycle arrest in the G1 phase significantly increased in the photoaging group, while the expression levels of p53 and p21 increased and the level of type I collagen decreased. All of the findings confirm the successful construction of the photoaging model.

The regulatory role of RNA m^6^A methylation modification in UV‐induced DNA damage response was first discovered in 2017 (Xiang et al., 2017). Based on the assumption that photoaging is essentially a form of UV damage, we postulated that RNA m^6^A methylation modification may also play a regulatory role in controlling photoaging. Our study found that total RNA m^6^A levels were reduced in photoaged cells, and the mRNA and protein expression levels of methyltransferase METTL14 were also decreased. These findings indicate that METTL14‐mediated RNA m^6^A methylation modification regulates the occurrence and development of photoaging. Furthermore, the overexpression of METTL14 was shown to alleviate UVB‐induced photoaging, suggesting the photoprotective effects of this protein. Several studies suggest that m^6^A methylation plays a crucial role in cellular senescence (Sun et al., 2022; Wu et al., 2020). Recent research has shown that UVB induces the degradation of METTL14 through autophagy in HaCat cells, leading to a decrease in overall m^6^A levels. And, overexpression of METTL14 can restore the levels of m^6^A and enhance DNA damage repair (Yang et al., 2021). These results complement our study and serve as a strong foundation for future research.

The initial findings of our study revealed a decrease in RNA m^6^A modification levels in photoaged HDFs, and we further discovered a reduction in METTL14 levels. Subsequently, our research focused on the downstream molecules of METTL14, inadvertently overlooking the cause of the METTL14 decrease. The effects of UV on DNA, RNA, and protein expression are highly intricate, involving direct photochemical actions and photosensitization, the latter predominantly induced by endogenous photosensitizers (EPs). These EPs consist of small molecules (vitamins, amino acids, cofactors, and metabolites from differentiated cells) and macromolecules (proteins, nucleic acids, polysaccharides) that are present in biological structures at specific concentrations and locations. The formation of cyclobutane pyrimidine dimers (CPDs) in the genome could impact the transcription and translation of RNA. Moreover, EPs can affect lysosomal function post‐UV irradiation, further influencing the autophagy process and thereby regulating cellular gene expression and metabolism (Bastos et al., 2023).

Previous research indicates that METTL14 interacts with DGCR8 and modulates pri‐miR‐126 via m^6^A‐dependent mechanisms (Ma et al., 2017). Our research affirms that METTL14 additionally affects the association of DGCR8 with the m^6^A position on pri‐miR‐100, thereby affecting its subsequent processing into mature miR‐100‐3p. Even though miR‐100‐3p has not yet been linked to cellular senescence, our findings show that it is a downstream target of METTL14 and has the potential to reverse its photoprotective impact. Moreover, we have discovered that miR‐100‐3p represses ERRFI1 mRNA translation, which results in a downregulation of ERRFI1 protein expression. ERRFI1, also known as Mitogen‐Inducible Gene 6 Protein or Mig‐6, is an adaptor protein that can be stimulated by mitosis, endocrine, and stress signals (Xu et al., 2005). While most studies have focused on the role of ERRFI1 in cancer research, where it is thought of as a tumor suppressor gene due to its low expression levels in different cancer types like lung, breast, and thyroid cancer (Li et al., 2012; Lin et al., 2011; Wendt et al., 2015), limited research has been conducted on its role in aging. In 2013, researchers discovered that ERRFI1 expression was elevated in senescent human diploid fibroblasts, and overexpression of ERRFI1 in normal human embryonic lung diploid fibroblasts induced premature senescence by impeding the EGFR/ErbB signaling pathway (Xie et al., 2013). A year later, another study verified that ERRFI1 expression was upregulated in senescent human diploid fibroblasts, and it regulated the phosphorylation of retinoblastoma protein (Rb), a cell senescence‐related protein (Milewska & Kolch, 2014). Our results are consistent with these studies and further illustrate the regulatory role of ERRFI1 in aging.

This study is the first to confirm that RNA m^6^A levels are reduced in UVB‐induced photoaged cells and that there is a concurrent decrease in METTL14 expression. Furthermore, overexpression of METTL14 has demonstrated photoprotective effects. There has been no prior research on the regulation of pri‐miR‐100 by the METTL14 methyltransferase through the m^6^A modification pathway. Our research is pioneering at the molecular level in discovering that, during photoaging, the methyltransferase METTL14 affects the m^6^A sites on pri‐miR‐100, thereby influencing the further processing of pri‐miR‐100 into mature miR‐100‐3p. This process, through the miR‐100‐3p–ERRFI1 axis, impacts the regulation of photoaging in human dermal fibroblasts. This finding holds promise for providing new targets for the prevention and treatment of skin photoaging in the future.

## 4 CONCLUSION

In conclusion, our research highlights that the methyltransferase METTL14 is downregulated in a UVB‐induced skin fibroblast photoaging model, while its overexpression exhibits a photoprotective effect. METTL14 performs a crucial role in mediating m^6^A methylation modification to regulate pri‐miR‐100, which directly influences the subsequent processing of miR‐100‐3p. Furthermore, our study has identified the key role of METTL14 in regulating skin fibroblast photoaging through the miR‐100‐3p/ERRFI1 pathway.

## 5 MATERIALS AND METHODS

### 5.1 Cell culture

Fibroblast cells were isolated from the skin of healthy adult males' foreskins. Cells at passages 2–7 were utilized for subsequent experimentation. This section of the study was granted ethical approval by the Ethics Committee of the Skin Disease Hospital, Chinese Academy of Medical Sciences (Approval No. 2022‐KY‐035). The cells were maintained in culture under standard conditions, specifically cultured within DMEM that had been supplemented with 10% FBS, and subsequently incubated at a temperature of 37°C whilst being exposed to an atmosphere containing 5% CO_2_.

### 5.2 Cell photoaging model

When the confluent rate of HDFs reached 60%–80%, the complete medium was replaced by a thin layer of PBS and irradiated with UVB (30 mJ/cm^2^) using a UVB cell irradiator (Shanghai SIGMA High‐Tech Co., Ltd., China). The irradiated cells were immediately treated with DMEM supplemented with 10% FBS and continuously cultured in a CO_2_ incubator at 37°C for an additional 24 h before subsequent experimentation. The control group cells were maintained under identical conditions without UVB irradiation.

### 5.3 Cell infection and transfection

The METTL14 cDNA sequence was amplified and subsequently subcloned into a lentiviral vector (Genechem, China) for the purpose of obtaining stable expression of METTL14 in HDFs cells. HDFs cells were then infected with recombinant lentiviruses containing the METTL14 construct (LV‐METTL14), followed by selection with puromycin (2 μg/mL) over a period of 2 weeks.

The HDFs were transfected with small‐interfering RNAs (siRNAs) targeting METTL14 (siMETTL14) and the negative control (siNC) (GenPharma, China) using siRNA‐mate (GenePharma, China). HDFs were transfected with miR‐100‐3p mimics, inhibitors, and their negative control (mimicNC, inhibitorNC) (RiboBio, China) using riboFECT™ CP transfection kit (RiboBio, China). The HDFs cells were transfected with an overexpression plasmid containing ERRFI1 or alternatively, a blank vector (Genechem, China), utilizing Lipofectamine 3000 (Invitrogen, USA) for the transfection process.

### 5.4 Senescence‐associated β‐galactosidase (SA‐β‐gal) assay

To evaluate cellular senescence, the SA‐β‐gal staining assay was employed to detect any evidence of aging. Specifically, cells that had been exposed to UVB radiation for a period of 24 h were subjected to SA‐β‐gal staining following the manufacturer's instructions provided in the Senescence β‐Galactosidase Staining Kit (Beyotime, China).

### 5.5 RNA isolation and RT‐qPCR

Total RNA was extracted from the samples using TRIzol (Invitrogen, USA) according to the manufacturer's protocol. The miRNA 1st strand cDNA synthesis kit (Accurate Biology, China) and Premix pro Taq HS qPCR Kit II (Accurate Biology, China) were employed for large‐scale screening of mature miRNAs, while detection of miR‐100‐3p relied on the miRNA 1st strand cDNA synthesis kit (by stem‐loop) (Vazyme, China) and miRNA universal SYBR qPCR master mix (Vazyme, China). For mRNA and pri‐miRNA analyses, HiScript III All‐in‐One RT superMix perfect for qPCR and ChamQ SYBR qPCR master mix (without ROX) (both from Vazyme, China) were used, respectively. Tsingke Biotech (China) synthesized all primers, and their specific sequences are provided in the Appendix S1. Expression levels were calculated using the $2^{-\Delta \Delta C_{t}}$ method, and GAPDH or U6 served as reference genes for normalization. The sequence of primers used in this study can be found in Appendix S1.

### 5.6 Western blot analyses

The total proteins were extracted from cells with radioimmunoprecipitation assay (RIPA) lysis buffer mixed with PMSF, protein inhibitors, and phosphatase inhibitors (Beyotime, China) and denatured by SDS‐PAGE loading buffer (Solarbio, China). An equal quantity of protein was separated using smartPAGE™ precast protein gel plus (4%–20%, Bis‐Tris) (Smart‐Lifesciences, China) in MOPS Running Buffer (Smart‐Lifesciences, China) and transferred to polyvinylidene difluoride (PVDF) membranes (Millipore, USA) in NcmBlot Rapid Transfer Buffer (NCM Biotech, China). The membranes were blocked with 5% nonfat dry milk (Beyotime, China) for 1.5 h and then incubated with primary antibodies: METTL14 polyclonal antibody (26158‐1‐AP, 1:1000, Proteintech, China), p21 polyclonal antibody (10355‐1‐AP, 1:1000, Proteintech, China), p53 polyclonal antibody (10442‐1‐AP, 1:1000, Proteintech, China), FTO polyclonal antibody (27226‐1‐AP, 1:1000, Proteintech, China), collagen type I polyclonal antibody (14695‐1‐AP, 1:1000, Proteintech, China), ERRFI1 polyclonal antibody (11630‐1‐AP, 1:1000, Proteintech, China), and GAPDH polyclonal antibody (10494‐1‐AP, 1:20000, Proteintech, China). Proteins were then incubated by anti‐rabbit IgG, HRP‐linked antibody #7074 (CST, USA) for 1.5 h and detected by an enhanced chemiluminescence system (ECL) reagent (Biosharp, China).

### 5.7 RNA m^6^A content assays

RNA isolation was performed using the TRIzol method (Invitrogen, USA) in accordance with the provided instructions. The dot‐blot assay was conducted following the outlined protocol present within the bio‐protocol database. In brief, 125/250/500 ng of the extracted RNA were deposited onto a Hybond‐N+ membrane, followed by cross‐linking via exposure to UV irradiation. To serve as a loading control, methylene blue was used to interact with RNA, and images were captured. Following washing, the membrane was blocked and then incubated overnight at 4°C with an anti‐m^6^A antibody (ab284130, 1:500, Abcam, UK). Thereafter, the membrane was treated with an anti‐rabbit IgG, HRP‐linked antibody #7074 (CST, USA) for 1.5 h and detected utilizing an enhanced chemiluminescence system (ECL) reagent (Biosharp, China). Finally, Hyperfilm ECL (Bio‐Rad) was utilized to expose the membrane, and images were subsequently captured.

### 5.8 Cell proliferation assays

For the purpose of evaluating the impact of UVB radiation on cell viability, a standardized protocol was employed wherein cells were seeded at equal densities in 35‐mm cell culture dishes. Cultures were incubated until they reached a confluence of 60%–70%, after which they were exposed to UVB radiation. Following a 24‐h post‐exposure period, cell viability was assessed using the CCK‐8 assay reagent (Vazyme, China) in accordance with the instructions. The results obtained were subsequently presented as a percentage relative to the control group.

### 5.9 Cell cycle assay

In order to analyze the cell cycle, cells were subjected to irradiation and harvested after 24 h. The harvested cells were processed by fixing with 70% ethanol, followed by incubation with RNase A and propidium iodide using the Cell Cycle and Apoptosis Analysis Kit (Beyotime, China) in accordance with the provided instructions. The obtained samples were subjected to analysis of cell cycle distribution utilizing flow cytometry (BD FACSVerse, USA), which was equipped with Modi Fit LT v5.0 software for data analysis. Quantification of the percentages of cells in G0/G1, S, and G2/M phases was conducted, and comparisons were made between groups.

### 5.10 Luciferase reporter assay

The predicted miR‐100‐3p binding sites were integrated into the pmirGLO luciferase vector (Pinuofei, China). Co‐transfection with either miR‐100‐3p mimics or their respective control counterparts (mimicNC) was conducted in 293 T cells utilizing Lipofectamine 3000 obtained from Invitrogen, USA. The relative luciferase activity was determined via application of the dual‐luciferase reporter assay system manufactured by Promega, located in USA, and subsequently normalized to Renilla luciferase activity.

### 5.11 RNA immunoprecipitation and methylated RNA immunoprecipitation

RNA immunoprecipitation (RIP) assays were conducted using the RNA immunoprecipitation kit (GenSeq, China) in accordance with the manufacturer's instructions. The cells were lysed using RIP lysis buffer and incubated overnight at 4°C with magnetic beads conjugated with anti‐DGCR8 antibody (Abcam, UK) or immunoglobulin G (IgG). Following incubation, the beads underwent treatment with proteinase K and shaking to eliminate any associated protein. The resulting coprecipitated RNAs were then isolated and subjected to RT‐qPCR using pri‐miR‐100 primers, which were subsequently normalized against input.

Methylated RNA immunoprecipitation (MeRIP) was performed using the m^6^A MeRIP kit (GenSeq, China) as per the manufacturer's instructions. RNAs were fragmented chemically, followed by incubation with magnetic beads conjugated with m^6^A antibody (Abcam, UK) for immunoprecipitation. The enrichment of m^6^A‐containing RNA was subsequently analyzed using RT‐qPCR and normalized to input.

## AUTHOR CONTRIBUTIONS

Dr Lihao Chen and Yu Hu performed the majority of the experiments, analyzed data, and prepared the manuscript. Dr Min Zhang, Jing Ma, Lihao Liu, and Zhuohong Xu isolated human dermal fibroblasts and performed a small part of RT‐qPCR and Western blot experiments. Professor Kun Chen, Heng Gu, and Jiaan Zhang supervised the project.

## CONFLICT OF INTEREST STATEMENT

The authors declare no competing financial interests.

## Supporting information


Appendix S1.

## Data Availability

The datasets [GSE119009] for this study can be found in the [GEO database] [https://www.ncbi.nlm.nih.gov/geo/query/acc.cgi?&acc=GSE119009].
