# Supplementary material for: METTL14 affects UVB‐induced human dermal fibroblasts photoaging via miR‐100‐3p biogenesis in an m6A‐dependent manner
Source: Aging Cell. 2024 Feb 21;23(5):e14123. doi: 10.1111/acel.14123 (PMC11113260; doi:10.1111/acel.14123)
Supplement: Supplementary file 1 — Appendix S1. [file ACEL-23-e14123-s001.docx]

**Primers used in this study**

| **Primer name** | **sequence（5’ – 3’）** |
| --- | --- |
| GAPDH-Forward | GGTGGTCTCCTCTGACTTCAA |
| GAPDH-Reverse | GTTGCTGTAGCCAAATTCGTTGT |
| METTL3-Forward | CCAGCACAGCTTCAGCAGTTCC |
| METTL3-Reverse | GCGTGGAGATGGCAAGACAGATG |
| METTL14-Forward | GTTGGAACATGGATAGCCGC |
| METTL14-Reverse | CAATGCTGTCGGCACTTTCA |
| METTL16-Forward | ACAGAAGACACTCCTGATGG |
| METTL16-Reverse | TTAACAGAACTAGGCGGAGG |
| WTAP-Forward | TTCCCAAGAAGGTTCGATTG |
| WTAP-Reverse | TGCAGACTCCTGCTGTTGTT |
| FTO-Forward | AGAATGTCTGTGACGATGTGG |
| FTO-Reverse | GCACTTTCTGTATCGATTGCC |
| ALKBH5-Forward | TGAGCACAGTCACGCTTCCC |
| ALKBH5-Reverse | TCCGTGTCCTTCTTTAGCGACTC |

| **Primer name** | **sequence（5’ – 3’）** |
| --- | --- |
| U6-Forward | CTCGCTTCGGCAGCACA |
| U6-Reverse | AACGCTTCACGAATTTGCGT |
| hsa-miR-100-3p(polyA) | CCAGCTTGTATCTATAGGTATG |
| hsa-miR-100-3p(stem-loop) | GTCGTATCCAGTGCAGGGTCCGAGGTATTCGCACTGGATACGACCATACC |
| hsa-miR-100-3p(stem-loop forward) | GCGCGGAAGCTTGTATCTATA |
| hsa-miR-100-3p(stem-loop reverse) | AGTGCAGGGTCCGAGGTATT |
| hsa-pri-miR-100-Forward | GAGTGGGACGAAGTCCTTTCC |
| hsa-pri-miR-100-Reverse | TTCGGATCTACGGGTTTGTGG |
| ERRFI1-Foward | GACCCACCGAAGATTAAGAAGG |
| ERRFI1-Reverse | GGTCTAGGAGGTATGGGAACTCT |

**Supplemental Figures**

**
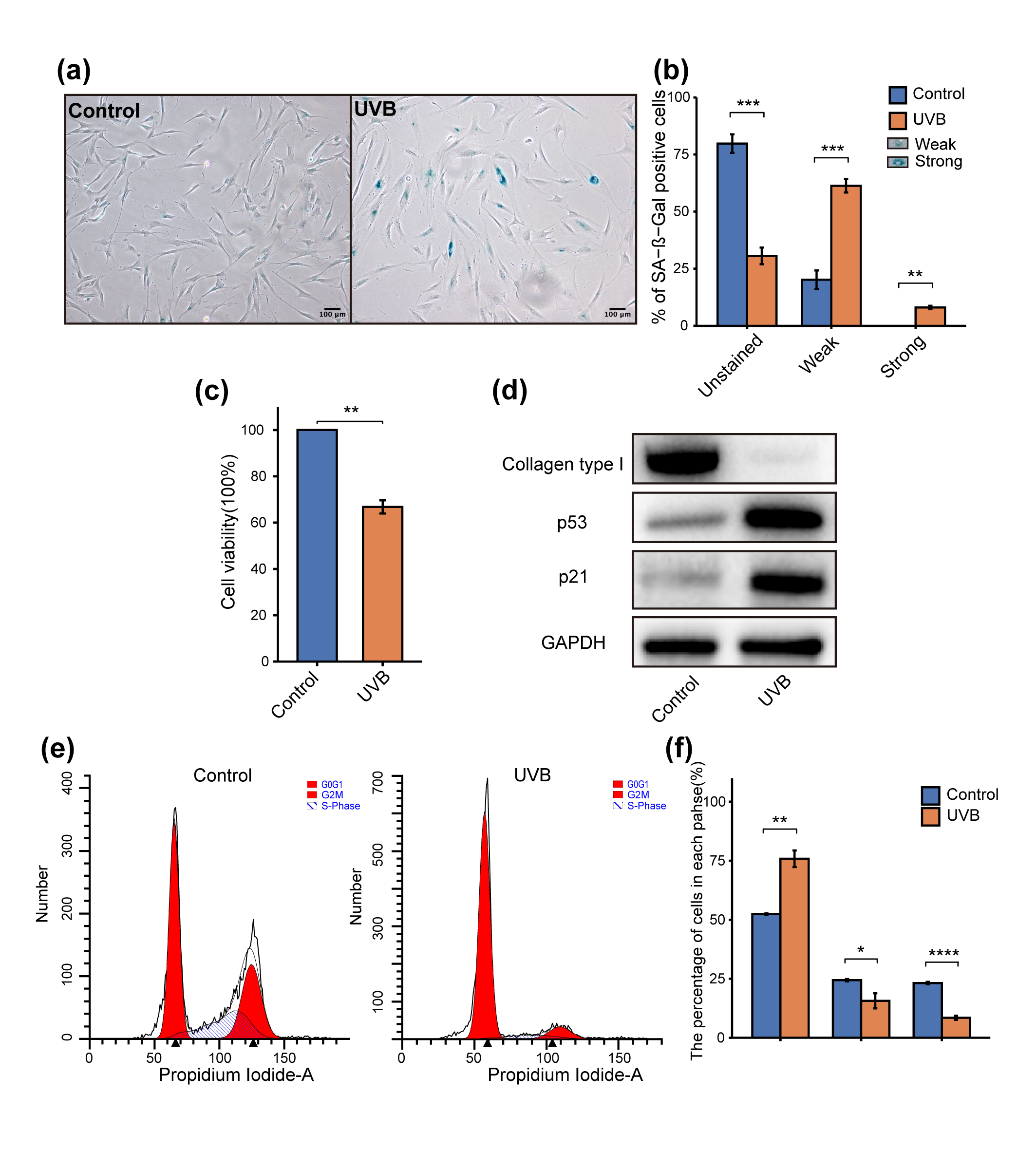
**

**Figure S1 Construction of UVB-induced HDFs photoaging model**

(a) HDFs were irradiated by UVB (30 mJ/cm2) to induce photoaging, and photoaged cells were identified by SA-β-Gal after 24h of irradiation. SA-β-Gal-positive cells were dyed blue under optical microscopy. (b) SA-β-Gal-positive cells were classified into two groups: strong and weak, and percentage of each group were shown in bar chart. (c) The effect of UVB on the cell viability of HDFs were evaluated by CCK-8. (d) Western blot analysis of collagen type I, p53 and p21 expression after 24h of UVB irradiation. (e, f) Cell cycle analysis was carried out 24h post-irradiation by flow cytometry. All data are the mean ± SD. *p < 0.05, **p < 0.01, ***p < 0.001, ****p < 0.0001. The significance of differences was determined with Student's t test.

**
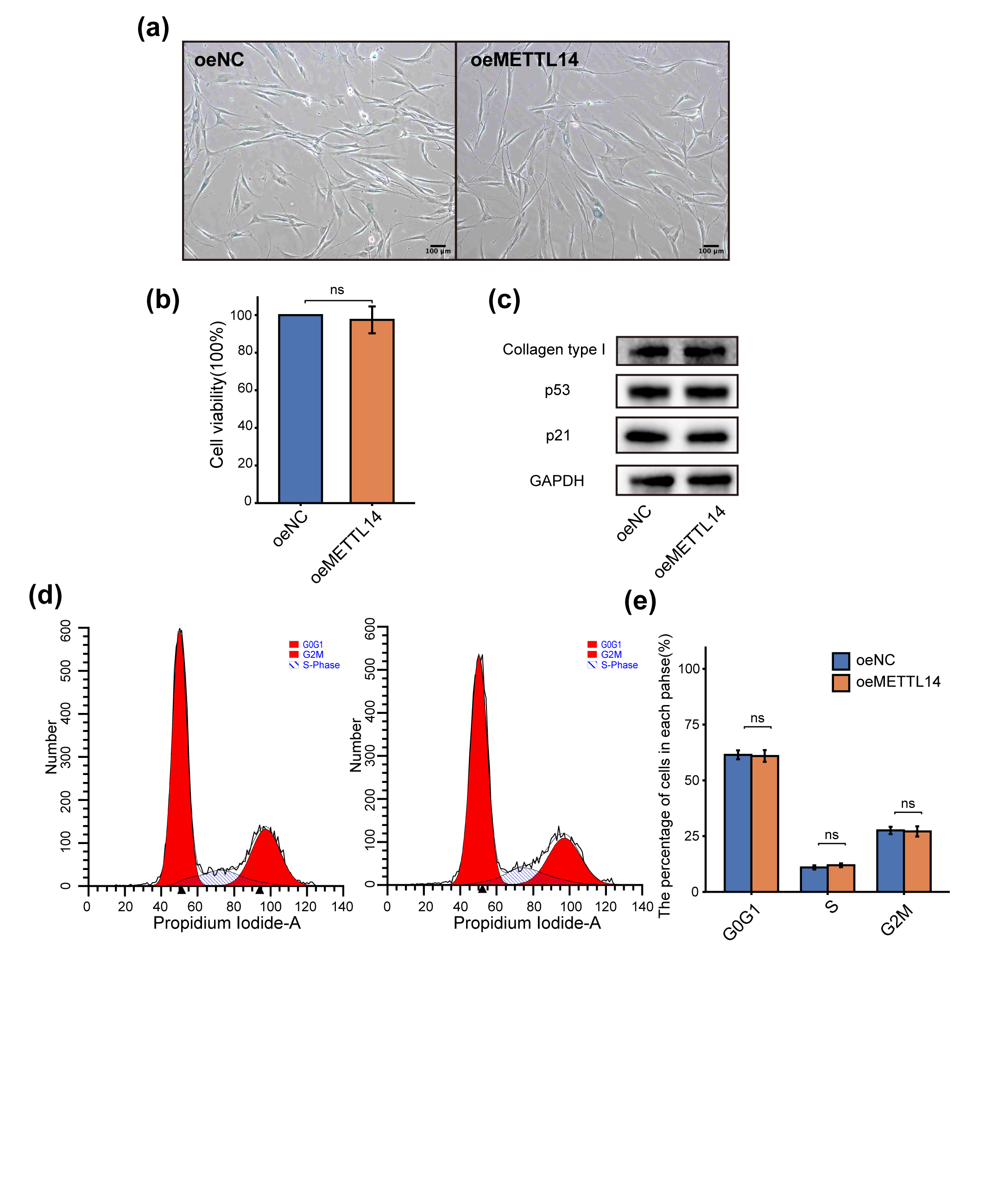
**

**Figure S2 Overexpression of METTL14 had no effects on HDFs**

(a) HDFs of oeNC and oeMETTL14 were stained by SA-β-Gal. (b) The effect of UVB on the cell viability of HDFs were evaluated by CCK-8. (c) Western blot analysis of collagen type I, p53 and p21 expression after 24h of UVB irradiation. (d, e) Cell cycle analysis was carried out 24h post-irradiation by flow cytometry. All data are the mean ± SD. The significance of differences was determined with Student's t test.

**
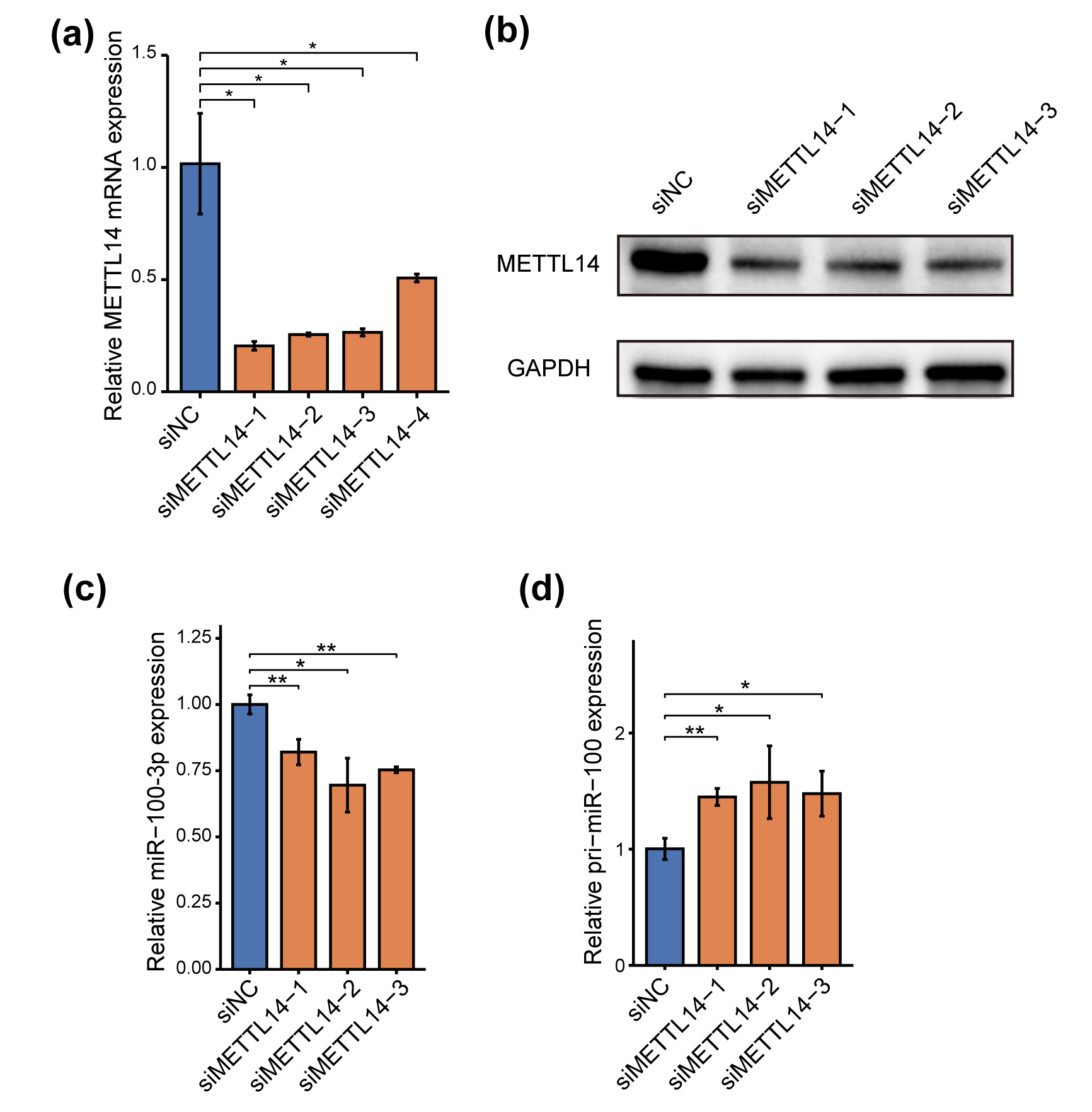
**

**Figure S3 Expression of miR-100-3p and pri-miR-100 after knockdown of METTL14**

(a, b) RT-qPCR and western blot of METTL14 in knockdown of METTL14 (siMETTL14) by siRNAs. (c) RT-qPCR of miR-100-3p in siMETTL14 and siNC HDFs. (e) RT-qPCR of pri-miR-100 in siMETTL14 and siNC HDFs. Cell cycle analysis was carried out 24h post-irradiation by flow cytometry. All data are the mean ± SD. *p < 0.05, **p < 0.01. The significance of differences was determined with Student's t test.

**
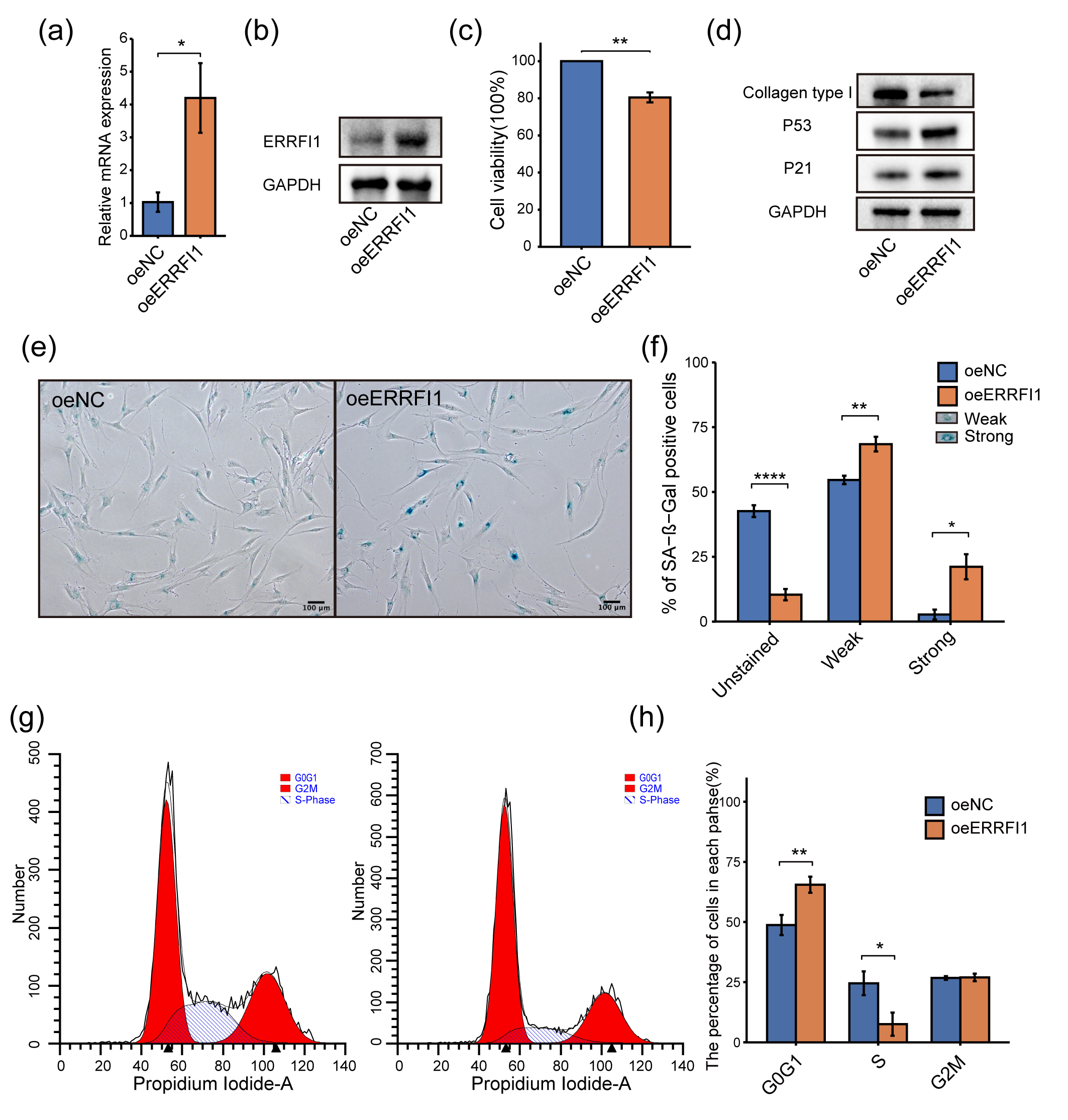
**

**Figure S4 Overexpression of ERRFI1 induced cellular senescence in HDFs**

(a, b) RT-qPCR and western blot of ERRFI1 in overexpression of ERRFI1 (oeERRFI1) by an overexpression plasmid containing ERRFI1 or a blank vector in HDFs. (c) CCK-8 was used to determine the cell viability of oeERRFI1 and oeNC HDFs. (d) Western blot analysis of collagen type I, p53 and p21 expression of oeERRFI1 and oeNC HDFs. (e) SA-β-Gal was used to identify senescent cells of oeERRFI1 and oeNC HDFs. SA-β-Gal-positive cells were dyed blue under optical microscopy. (f) SA-β-Gal-positive cells were classified into two groups: strong and weak, and percentage of each group were shown in bar chart. (g, h) Cell cycle analysis of oeERRFI1 and oeNC HDFs by flow cytometry. All data are the mean ± SD. *p < 0.05, **p < 0.01, ***p < 0.001, ****p < 0.0001. The significance of differences was determined with Student's t test.
